# Acute kidney injury after COVID-19 vaccines: a real-world study

**DOI:** 10.1080/0886022X.2022.2081180

**Published:** 2022-06-09

**Authors:** Huiting Luo, Xiaolin Li, Qidong Ren, Yangzhong Zhou, Gang Chen, Bin Zhao, Xuemei Li

**Affiliations:** a Nephrology Department, Peking Union Medical College Hospital, Peking Union Medical College, Chinese Academy of Medical Sciences, Beijing, China; b State Key Laboratory of Bioactive Substance and Function of Natural Medicines, Institute of Materia Medica, Peking Union Medical College Hospital, Peking Union Medical College, Chinese Academy of Medical Sciences, Beijing, China; c Pharmacy Department, Peking Union Medical College Hospital, Peking Union Medical College, Chinese Academy of Medical Sciences, Beijing, China

**Keywords:** COVID-19, vaccination, acute kidney injury, VAERS database

## Abstract

**Background:**

Acute kidney injury (AKI), a rare adverse event, cannot be ignored as millions of doses of coronavirus disease 2019 (COVID-19) vaccinations. We aimed to investigate the occurrence of post-vaccine AKI reported to the Vaccine Adverse Event Reporting System (VAERS).

**Methods:**

After data mapping from December 2020 to June 2021, we summarized demographic and clinical features and outcomes of reported cases from three vaccines (Pfizer-BNT, MODERNA, and JANSSEN). The Bayesian and nonproportional analyses explored the correlations between COVID-19 vaccines and AKI.

**Results:**

We identified 1133 AKI cases. Pfizer-BNT appeared to have a stronger AKI correlation than MODERNA and JANSSEN, based on the highest reporting odds ratio (ROR = 2.15, 95% confidence interval = 1.97, 2.36). We observed the differences in ages, comorbidities, current illnesses, post-vaccine AKI causes, and time to AKI onset (all *p*＜.05) among three vaccines. Most patients are elderly, with the highest age in MODERNA (68.41 years) and lowest in JANSSEN (59.75 years). Comorbidities were noticed in 58.83% of the cases and active infections in over 20% of cases. The leading cause of post-vaccine AKI was volume depletion (40.78%), followed by sepsis (11.74%). Patients in Pfizer-BNT had the worst outcome with 19.78% deaths, following 17.78% in MODERNA and 12.36% in JANSSEN (*p =* .217). The proportion of patients on dialysis was higher in JANSSEN than in Pfizer-BNT and MODERNA (14.61% vs. 6.54%, 10.62%, *p =* .008).

**Conclusion:**

AKI could occur after the COVID-19 vaccines, predominantly in elderly patients. However, the causality needs further identification.

## Introduction

Globally, as of 17 December 2021, more than 271 million confirmed cases of coronavirus disease 2019 (COVID-19) and over 5 million COVID-19-related deaths had been reported by the world health organization (WHO) [1]. The vaccination campaign is urgently ongoing worldwide to contain the pandemic by reducing morbidity and mortality. The US Food and Drug Administration (FDA) authorized two messenger RNA-based vaccines (Pfizer-BioNTech and Moderna) in December 2020 and an adenovirus vector-based vaccine (Janssen) in February 2021 for emergency use. Aside from nonsevere transient local and systemic reactions, those vaccines have been proven safe and effective by large clinical trials [2–4]. As of December 2021, more than 493 million doses have been administrated in the United States [5].

In the meantime, reports of rare adverse events (AEs) are emerging in the short interval of vaccine injection, in terms of *de novo* or recurrent kidney diseases, such as minimal change disease (MCD) [6], anti-neutrophil cytoplasmic antibody-associated glomerulonephritis [7], IgA nephropathy [8], membranous nephropathy [9], anti-glomerular basement membrane nephritis [10], acute tubulointerstitial nephritis [11], thrombotic thrombocytopenic Purpura [12], and thrombotic microangiopathy [13]. Interestingly, acute kidney injury (AKI) is accompanied in most such conditions, as exemplified by the finding that AKI was observed in most published cases of MCD and over 70% of glomerulonephritis [14]. Moreover, a proportion of the reported patients progressed to end-stage kidney disease (ESKD), requiring renal replacement therapy.

AKI is associated with poor outcomes due to the development of chronic kidney disease (CKD) and ESKD and a higher mortality rate [15,16]. Individuals with old age, preexisting CKD, or other comorbidities are at risk for COVID-19 infection, and they are also more likely to develop AKI after vaccination [16,17]. Therefore, post-marketing surveillance is needed for such increasing concerns. This study aimed to investigate the demographics, clinical features, and outcomes of AKI after COVID-19 vaccines across different vaccine manufacturers in the real world, relying on reports from the Vaccine Adverse Event Reporting System (VAERS).

## Methods

### Database description

VAERS is a self-reporting system for post-vaccination AEs jointly administered by the Centers for Disease Control and Prevention and the FDA [18]. VAERS acts as an early warning system to test for possible safety issues with US-licensed vaccines by collecting information about AEs after vaccination.

VAERS data contains the following files: VAERSDATA.CSV (including reports and patient information), VAERSVAX.CSV (including vaccine information), and VAERSSYMPTOMS.CSV (including the AE information). Patient information includes demographic data, coexisting current illnesses, and comorbidities. Vaccine information includes vaccine manufacturers. AE information includes vaccination date, symptom onset date, AE description, and prognosis. We defined the onset of AEs by calculating the interval between vaccination date and symptom onset date.

We described all reports to VAERS submitted for persons vaccinated with COVID-19 Vaccines during the period December 2020 through June 2021.

### Adverse event and drug identification

AKI was based on Medical Dictionary for Regulatory Activities (MedDRA, version 24.0) at the preferred term (PT) level. We considered the following PTs as related to AKI: ‘acute kidney injury [10069339]’, ‘subacute kidney injury [10081980]’, ‘blood creatinine increased [10005483]’, ‘blood urea abnormal [10005846]’, ‘glomerular filtration rate decreased [10018358]’, ‘renal impairment [10062237]’, ‘oliguria [10030302]’, ‘anuria [10002847]’, ‘dialysis [10061105]’, ‘proteinuria [10037032]’, ‘renal tubular injury [10078933]’, ‘nephropathy toxic [10029155]’, ‘nephritis allergic [10029120]’, and ‘tubulointerstitial nephritis [10048302]’.

Two physicians (Chen G and Zhou Y) who specialized in nephrology with more than 7 years of experience independently analyzed the descriptions in the database to ensure the reliability of the AKI diagnosis. They also viewed the coexisting current illnesses and comorbidities described in natural language in VAERS. If they disagreed with the judgment of the description, they would turn to a professor (Li X) with more than 15 years of experience in nephrology.

### Data mining

Based on the basic principles of Bayesian analysis and nonproportional analysis, the reporting odds ratio (ROR), the proportional reporting ratio (PRR), the Bayesian confidence propagation neural network (BCPNN), and the multi-item gamma Poisson shrinker (MGPS) algorithms were used to study the relationship between COVID-19 Vaccines and AKI. Table 1 lists the equations and criteria for the four algorithms.

**Table 1.** Summary of major algorithms used for signal detection.

Algorithms	Equation	Criteria
ROR	ROR=(a/b)/(c/d)	95% CI > 1, *N* ≥ 2
	$95\%CI=e^{ln(\mathrm{ROR})\pm 1.96{\left(1/a+1/b+1/c+1/d\right)}^{0.5}}$	
PRR	PRR=(a/(a+c))/(b/(b+d))	PRR ≥ 2, χ^2^≥4, N ≥ 3
	χ2=Σ((O−E)2/E); (O=a, E=(a+b)(a+c)/(a+b+c+d))	
BCPNN	$\text{IC}={\;\text{log}\;}_{2}a\left(a+b+c+d\right)/\left(\left(a+c\right)\left(a+b\right)\right)$	IC025 > 0
	$IC025=e^{\;\text{ln}\;(\text{IC})-1{.96(1/a+1/b+1/c+1/d)}^{\wedge 0,5}}$	
MGPS	EBGM=a(a+b+c+d)/((a+c)(a+b))	EBGM05 > 2, *N* > 0
	${\text{EBGM}05=e}^{\;\text{ln}\;(\text{EBGM})-1{.64(1/a+1/b+1/c+1/d)}^{\wedge }{}^{0.5}}$	

a: number of reports containing both the suspect drug and the suspect adverse drug reaction; b: number of reports containing the suspect adverse drug reaction with other medications (except the drug of interest); c: number of reports containing the suspect drug with other adverse drug reactions (except the event of interest); d: number of reports containing other medications and other adverse drug reactions; ROR: reporting odds ratio; CI: confidence interval; N: the number of co-occurrences; PRR: proportional reporting ratio; χ^2^: chi-squared; BCPNN: Bayesian confidence propagation neural network; IC: information component; IC025: the lower limit of the 95% two-sided CI of the IC; MGPS: multi-item gamma Poisson shrinker; EBGM: empirical Bayesian geometric mean; EBGM05: the lower 90% one-sided CI of EBGM.

### Statistical analysis

We conducted a descriptive analysis of AKI cases. The chi-square test was used to compare the proportions of each descriptive variable in each group. If continuous variables were normally distributed, means were compared using a one-way analysis of variance. Differences were considered significant if *p* < .05. Statistical analysis was performed by SPSS, version 21 (IBM Inc., NC, USA).

## Results

### Demographics

A total of 1133 cases of AKI after COVID-19 vaccinations were retrieved, including 616 cases with the Pfizer-BNT, 429 with the MODERNA, and 88 with the JANSSEN. Patient demographics are summarized in Table 2. We detected no statistical significance concerning gender among the three groups. We observed the differences in age among three groups (*p* < .001), with the highest in MODERNA (68.41 ± 16.14 years) and the lowest in JANSSEN (59.75 ± 15.63 years). Most of the affected cases were reported in old-aged groups among Pfizer-BNT and MODERNA, while the middle-aged group (45–64 years, 42.86%) were most reported in JANSSEN. Interestingly, AKI at <18 years of age had only 12 patients in the Pfizer-BNT group, as it was the only one approved for vaccination in the 12–18 years old population. AKI cases were most commonly reported in Illinois, California, and Florida, mainly from March to May.

**Table 2.** Demographic data among patients with AKI following the COVID-19 vaccines sourced from the VAERS database (December 2020 to June 2021).

Demographic data	Pfizer-BNT	MODERNA	JANSSEN	*p*
Gender, available number	616	429	88	
Male, n (%)	313 (50.00)	235 (54.78)	42 (47.73)	.234
Female, n (%)	313 (50.00)			
Age, years	67.31 ± 17.47	68.41 ± 16.14	59.75 ± 15.63	<.001*
Age groups, available number	606	424	84	
<18, *n* (%)	12 (1.98)	0 (0.00)	0 (0.00)	<.001*
18–44, *n* (%)	56 (9.24)	47 (11.08)	15 (17.86)	
45–64, *n* (%)	145 (23.93)	81 (19.10)	36 (42.86)	
65–74, *n* (%)	160 (26.40)	119 (28.07)	18 (21.43)	
75–84, *n* (%)	147 (24.26)	122 (28.77)	10 (11.90)	
>85, *n* (%)	86 (14.19)	55 (12.97)	5 (5.95)	
Top 3 AKI reporting states, *n* (%)				
Illinois	32/627 (5.10)	37/433 (8.55)	13/89 (14.61)	–
California	38/627 (6.06)	31/433 (7.16)	5/89 (5.62)	–
Florida	17/627 (2.71)	25/433 (5.77)	5/89 (5.62)	–
Vaccine receiving date, *n* (%)				
December 2020	11/627 (1.75)	2/433 (0.46)	0/89 (0.00)	<.001*
January 2021	37/627 (5.90)	36/433 (8.31)	0/89 (0.00)	
February 2021	90/627 (14.35)	68/433 (15.70)	0/89 (0.00)	
March 2021	108/627 (17.22)	112/433 (25.87)	7/89 (7.87)	
April 2021	105/627 (16.75)	93/433 (21.48)	41/89 (46.07)	
May 2021	216/627 (34.45)	96/433 (22.17)	31/89 (34.83)	
June 2021	60/627 (9.57)	26/433 (6.00)	10/89 (11.24)	

COVID-19: coronavirus disease 19; AKI: acute kidney injury; VARES: vaccine adverse event reporting system.

### Clinical characteristics

We summarized the clinical characteristics in Table 3. There were significant differences in comorbidities (*p <* .001), coexisting current illnesses (*p =* .014), post-vaccine AKI causes (*p =* .008), and time to AKI onset (*p =* .008) among the three different COVID-19 vaccines. Comorbidities were reported in more than half of the cases (58.83%), and it was most common in the MODERNA group. Hypertension counted as the most common past disease in Pfizer-BNT (28.27%), MODERNA (34.87%), and JANSSEN (30.34%), respectively. It should be noted that chronic kidney diseases were also a relatively common past disease for Pfizer-BNT (20.77%), MODERNA (21.02%), and JANSSEN (17.98%). Current infection, including pneumonia, urinary tract infection, and upper respiratory tract infection, was the most common coexisting active illness among the vaccinees who developed AKI. The leading post-vaccine AKI cause was volume depletion; Pfizer-BNT accounted for 34.13%, MODERNA accounted for 41.11%, and JANSSEN accounted for 48.31%. For other AKI causes, thrombotic microangiopathy occurred most often in JANSSEN (11.24%), compared with Pfizer-BNT (3.51%) and MODERNA (4.85%). AKI occurred earliest in MODERNA among all COVID-19 vaccines, with a median onset time of 10.61 ± 16.55 days (*p =* .008). AKI following the COVID-19 vaccines led to poor prognosis, with 19.78% death in the Pfizer-BNT group, 17.78% in MODERNA, and 12.36% in JANSSEN. 23.60% of cases of AKI concerning JANSSEN were admitted to the clinic visit, and 55.34% of cases of AKI concerning Pfizer-BNT visited the emergency room. Dialysis requirement rates after AKI were different among the three vaccine groups, with the highest in JANSSEN (14.61%) and the lowest in Pfizer-BNT (6.54%) (*p =* .008).

**Table 3.** Clinical characteristics among patients with AKI following the COVID-19 vaccines sourced from the VAERS database (December 2020 to June 2021).

Clinical characteristics	Pfizer-BNT (*n* = 627)	MODERNA (*n* = 433)	JANSSEN (*n* = 89)	*p*
Comorbidities, *n* (%)	327 (52.24)	292 (67.44)	57 (64.04)	<.001*
Hypertension, *n* (%)	177 (28.27)	151 (34.87)	27 (30.34)	.073
Diabetes, *n* (%)	105 (16.80)	102 (23.56)	19 (21.35)	.023
Chronic kidney diseases, *n* (%)	130 (20.77)	91 (21.02)	16 (17.98)	.807
Heart diseases, *n* (%)	117 (18.69)	102 (23.56)	16 (17.98)	.129
Asthma and COPD, *n* (%)	46 (7.35)	46 (10.62)	10 (11.24)	.132
Gastrointestinal diseases, *n* (%)	95 (15.18)	70 (16.17)	12 (13.48)	.791
Connective tissue diseases, *n* (%)	18 (2.88)	13 (3.00)	0 (0.00)	0.260
Anemia, *n* (%)	28 (4.47)	21 (4.85)	5 (5.62)	0.878
Coagulation disorders, *n* (%)	22 (3.51)	18 (4.16)	1 (1.12)	0.371
Neurological disorders, *n* (%)	71 (11.34)	58 (13.39)	8 (8.99)	0.402
Surgery or radiotherapy, *n* (%)	45 (7.19)	39 (9.01)	8 (8.99)	0.529
Cancer, *n* (%)	63 (10.06)	39 (9.01)	6 (6.74)	0.565
Coexisting active illnesses, *n* (%)	121 (19.30)	116 (26.79)	22 (24.72)	0.014*
Infection, *n* (%)	35 (5.58)	38 (8.78)	4 (4.49)	0.085
Pneumonia, *n* (%)	20 (3.19)	17 (3.93)	1 (1.12)	0.392
Urinary tract infection, *n* (%)	7 (1.12)	6 (1.39)	1 (1.12)	0.922
Upper respiratory tract infection, *n* (%)	0 (0.00)	2 (0.46)	1 (1.12)	0.088
Other infection, *n* (%)	7 (1.12)	16 (3.70)	2 (2.25)	0.018
Heart failure, *n* (%)	10 (1.59)	8 (1.85)	1 (1.12)	0.875
Other active illnesses, *n* (%)	105 (16.75)	99 (22.86)	20 (22.47)	0.036
Post-vaccine AKI causes				
Volume depletion, *n* (%)	214 (34.13)	178 (41.11)	43 (48.31)	0.008*
Nausea and vomiting, *n* (%)	101 (16.11)	89 (20.55)	21 (23.60)	0.077
Diarrhea, *n* (%)	57 (9.09)	45 (10.39)	9 (10.11)	0.771
Fever, *n* (%)	138 (22.01)	123 (28.41)	32 (35.96)	0.004*
Decreased appetite, *n* (%)	21 (3.35)	11 (2.54)	3 (3.37)	0.740
Sepsis, *n* (%)	70 (11.16)	54 (12.47)	9 (10.11)	0.730
Acute tubular necrosis, *n* (%)	5 (0.80)	5 (1.15)	2 (2.25)	0.435
Acute interstitial nephritis, *n* (%)	0 (0.00)	2 (0.46)	0 (0.00)	0.291
Glomerular nephritis, *n* (%)	10 (1.59)	10 (2.31)	2 (2.25)	0.686
Nephrotic syndrome, *n* (%)	1 (0.16)	0 (0.00)	1 (1.12)	0.149
Thrombotic microangiopathy, *n* (%)	22 (3.51)	21 (4.85)	10 (11.24)	0.005*
Pulmonary embolism, *n* (%)	17 (2.71)	14 (3.23)	3 (3.37)	0.861
Time to AKI onset, days	14.11 ± 19.99	10.61 ± 16.55	11.32 ± 13.03	0.008*
Clinic visit, *n* (%)	121 (19.30)	93 (21.48)	21 (23.60)	0.513
ER visit, *n* (%)	347 (55.34)	235 (54.27)	42 (47.19)	0.352
Hospitalization, *n* (%)	457 (72.89)	295 (68.13)	68 (76.40)	0.133
Length of stay, days	3.68 ± 5.97	3.54 ± 5.18	4.72 ± 7.15	0.305
Dialysis initiated, *n* (%)	41 (6.54)	46 (10.62)	13 (14.61)	0.008*
Death, *n* (%)	124 (19.78)	77 (17.78)	11 (12.36)	0.217
AKI onset to death, days	8.57 ± 15.16	9.50 ± 15.63	9.00 ± 11.57	0.915

COVID-19: coronavirus disease 19; AKI: acute kidney injury; VARES: vaccine adverse event reporting system; ER: emergency room.

### Disproportionality analysis and Bayesian analysis

AKI signals were detected for all three COVID-19 Vaccines based on the criteria for the four algorithms, and the results are listed in Table 4. Pfizer-BNT was noteworthy for the relationship to AKI among all vaccines due to its statistically significant ROR (2.15 95% CI (1.97, 2.36)), PRR (2.15(290.75)), and EBGM (1.87(1.73)). At the same time, JANSSEN and MODERNA appeared to have a relatively weaker association with AKI than Pfizer-BNT, based on its ROR and IC.

**Table 4.** Signals in different Covid-19 vaccines with AKI.

Vaccine	*N*	ROR	PRR	IC	EBGM
		(95% two-sided CI)	(χ^2^)	(IC025)	(EBGM05)
JANSSEN	89	1.04 (0.84, 1.28)	1.04 (0.12)	0.05 (0.04)	1.04 (0.87)
MODERNA	433	1.25 (1.13, 1.39)	1.25 (17.97)	0.27 (0.24)	1.21 (1.11)
Pfizer-BNT	627	2.15 (1.97, 2.36)	2.15 (290.75)	0.9 (0.82)	1.87 (1.73)

COVID-19: coronavirus disease 19; AKI: acute kidney injury; *N*: the number of reports of COVID-19 vaccine-associated AKI; ROR: reporting odds ratio; CI: confidence interval; PRR: proportional reporting ratio; χ^2^: chi-squared; IC: information component; EBGM: empirical Bayes geometric mean.

## Discussion

The COVID-19 vaccines are generally considered safe, and AKI post-vaccination is deemed a rare adverse event. AKI was not reported in clinical trials [3–5] and was limited to several case reports [6–14]. Our previous real-world analysis suggested that renal and urinary disorders accounted for 0.46% of 3908 AEs until January 2021 [19]. Nonetheless, with the large-scale vaccination, the number of AKI cannot be underestimated. Real-world research demonstrated unique superiority for events with a very low incidence. To our knowledge, this study is the first and most extensive collection until recently to describe the occurrence of AKI after the COVID-19 vaccination in real-world practice based on the VAERS pharmacovigilance database, which is a self-reporting system that can serve as an early warning system for vaccine safety issues, including renal adverse effects [18]. Similarly, recent reports on the VAERS system have drawn attention to post-vaccine cerebrovascular events, myocarditis, and Guillain-Barré Syndrome [20–22]. We summarized 1133 AKI cases post COVID-19 vaccines and confirmed the AKI signal after the COVID-19 vaccines. About 71.4% of cases were reported between March and May, consistent with the number of people vaccinated, over 90% of whom were vaccinated [5]. Notably, 54.6% of cases were from the Pfizer-BNT group since the Pfizer-BNT is the most widely administered vaccine with a broader population (>12 years) [5].

The VAERS database found that 63.7% of patients were older than 65 years, and more than 50% had underlying diseases, such as hypertension and diabetes, followed by CKD. This is congruent with previous findings that AKI tended to be more likely to develop in the elderly (median age 63), males (57%), with comorbidities (i.e., diabetes 28%), or with CKD (26%) for the United States, a high-income country [23,24]. A meta-analysis indicated that age, gender, comorbidities, and CKD are risk factors for AKI [25]. One latest study noted that the risk of AKI increased 2-fold in the presence of diabetes with CKD [26]. This suggested the need to have an increased awareness of the possibility of post-vaccine renal AEs in the elderly or those with comorbidities. The coexisting active infections should also be emphasized, given that 42.1% of patients with sepsis developed concomitant AKI, and septic AKI was associated with greater severity of AKI and higher mortality [27]. Correspondingly, sepsis became the second leading cause of post-vaccination AKI in our study. Our unpublished data also suggested that people with coexisting current illnesses had an increased risk of AKI-associated mortality than those without when receiving COVID-19 mRNA vaccines. It is recommended that vaccinations may need to be administered more carefully in the presence of infection.

The most common causes of AKI in high-income countries are hypotension or shock (45%), followed by dehydration (39%) and sepsis (27%), whereas dehydration (46%), sepsis (39%), and hypotension or shock (38%) in low-income countries [24]. This discrepancy may be explained because most of the AKI was related to ICU hospitalizations in the former, while community-acquired infections predominantly caused AKI in the latter. Interestingly, in our study, post-vaccine AKI was mainly associated with volume depletion, consistent with the causes in low-income countries. Besides inadequate oral intake or vomiting, such volume depletion was usually due to fever in the database, which was among the most common AE following immunization [19]. In addition, the high rate of TMA should be alerted, particularly in the JANSSEN group (11.24%). The FDA suspended the JANSSEN vaccine interim and added a warning about its rare clotting events, such as cerebral venous sinus thrombosis, caused by the production of platelet-activating antibodies, similar to the autoimmune heparin-induced thrombocytopenia [28,29]. AKI following vaccines resulted in about 70% of hospitalizations and almost 20% of deaths in terms of outcomes. Moreover, the time from AKI to death is as short as approximately 10 days. It was indicated in another study that the average pooled AKI-related mortality rate was 23% but reached 49.4% in those requiring renal replacement therapy, primarily in hospital settings [30]. Those are indicative of rapid progression and unfavorable outcomes of AKI.

We noted that most AKI developed within two weeks after immunizations, keeping with previous studies [6–13]. However, we cannot establish a causal relationship between COVID-19 vaccinations and AKI. It was not uncommon that AKI or nephropathies could be caused by various vaccines, including flu, H1N1, and others [31–33]. Nonetheless, the incidence of COVID-19 vaccines related to AKI may be even higher, probably for the following reasons. First, as mass vaccination, people at risk of AKI will receive the COVID-19 vaccine but most likely will not receive vaccines such as influenza. Second, the mRNA vaccine has been developed and refined for nearly two decades, but it was not widely used clinically until only recently [34]. Compared with traditional vaccines, mRNA vaccine can elicit robust antibody- and cell-mediated immune responses, as demonstrated by its significantly higher neutralizing antibody titers and activated CD4^+^ and CD8^+^ T cells accompanied by increasing pro-inflammatory cytokines [35,36]. Moreover, it should be noted that potential risks of crossreactivity between SARS-CoV-2 and autoimmune target proteins could lead to immune activation that may aggravate, unmask, or incite autoimmune diseases [37,38]. Our results also imply that mRNA vaccines (Pfizer-BioNTech and Moderna) were more commonly associated with AKI than adenovirus vaccines (Janssen). Third, the immune response to the COVID-19 vaccine may mimic what happens in response to natural infection. The kidneys are not innocent bystanders during the involvement of COVID-19 disease. Up to 50% of hospitalized patients with COVID-19 presented AKI and higher mortality, especially critically ill patients (40–60%) [39,40]. The underlying mechanisms of COVID-19-associated AKI are likely multifactorial, including local and systemic inflammatory and immune responses, endothelial injury and hypercoagulability, and the renin-angiotensin system, direct viral infection [41].

Finally, there are some limitations to this study. First, the incidence rate cannot be calculated because the total number of patients receiving vaccinations is not provided in VAERS. We estimated the incidence of AKI to be 0.006% using the number of people who receive at least one dose of vaccine (18 million) as the denominator [5]. Second, incomplete or redundant data may lead to bias in the analysis. Since VAERS is a voluntary report system, patients with mild AKI may not be reported, leading to bias toward a disproportionately high ratio of severe AKI. Third, we could not profile the AKI staging or identify the risk factors since the critical data that impacted AKI, such as baseline creatinine, urine protein, medications, and socioeconomic status, were unavailable [24]. Fourth, causality could not be set up by investigating the AKI reports after vaccines; some reasons other than the vaccines could contribute to the AKI. Fifth, many important and detailed clinical data are not available, including renal pathology and the number of comorbidities in a patient. In addition, the long-term outcomes of AKI remain unknown due to the lack of patient follow-up.

In conclusion, post-vaccine renal AEs may require more attention in the elderly, those with comorbidities, and coexisting active diseases. Further on, patients with active infections need to be alerted to AKI after vaccination. We indicated an association with increased risk of AKI after COVID-19 immunization; however, the causality needs further identification.

## Data Availability

The data sets and resources analyzed during the current study are available from the corresponding author on reasonable request.
